# Accelerated Loss of Residual Kidney Function in Incremental Hemodialysis

**DOI:** 10.7759/cureus.78601

**Published:** 2025-02-06

**Authors:** Joana Medeiros, José Mário Bastos, Catarina Silva, Johanna Viana, Bárbara Ribeiro, Renata Carvalho, Rui Miguel Costa

**Affiliations:** 1 Nephrology Department, Hospital de Braga, Braga, PRT

**Keywords:** hemodialysis adequacy, incremental hemodialysis, renal urea clearance, residual kidney function, twice-weekly hemodialysis

## Abstract

Background

Incremental hemodialysis (iHD) involves gradually increasing dialysis dose as residual kidney function (RKF) declines. RKF has been identified as a strong predictor of patient survival. Given the importance of maintaining RKF in hemodialysis patients, our study aimed to analyze predictors of accelerated RKF loss in those undergoing iHD.

Methods

We retrospectively analyzed 37 incident patients who started twice-weekly iHD between March 2017 and May 2022. Inclusion criteria were age ≥18 years, Kru ≥3 ml/min/1.73 m^2^, urinary output ≥500 ml/day, and ability to comply with monthly interdialytic urine collection. Accelerated RKF loss was defined as a ≥25% decrease in Kru from baseline within three months.

Results

Of 30 patients with complete data, 30% (n=9) experienced accelerated RKF loss. These patients were older, had a higher prevalence of diabetes, more comorbidities, and lower baseline glomerular filtration rate (GFR). They also had more intradialytic complications and were more likely to have prescribed dry weight exceeding normohydrated weight by >1 kg. Multivariate analysis showed only baseline GFR below 7 ml/min/1.73 m^2^ independently predicted accelerated RKF loss.

Conclusions

Our study identifies baseline GFR below 7 ml/min/1.73 m^2^ as a key independent predictor of accelerated RKF loss in iHD patients. While factors such as older age, diabetes, higher comorbidity burden, and intradialytic complications were associated with accelerated RKF loss in univariate analysis, they were not significant in the multivariate model. These findings highlight the importance of careful patient selection and management in iHD programs, with particular attention to baseline GFR. By identifying patients at higher risk of rapid RKF decline, clinicians can implement targeted strategies to preserve RKF and potentially improve long-term outcomes.

## Introduction

Incremental hemodialysis (iHD) represents a paradigm shift in the initiation of renal replacement therapy for patients with end-stage kidney disease (ESRD). This approach involves starting dialysis with fewer sessions per week or shorter treatment times, gradually increasing the dialysis dose as residual kidney function (RKF) declines.

The preservation of RKF has been the driving impetus for iHD, alongside other potential benefits such as lower risk of hospitalizations, lower costs, tendency towards lower rates of vascular access complications, lower cardiovascular events and lower mortality [1-5].

RKF has been identified as a strong predictor of patient survival, with each 1 ml/min increase in renal urea clearance (Kru) associated with a 7% reduction in mortality risk [6]. Lower ultrafiltration requirements, better potassium clearance, reduced erythropoietin requirements and erythropoietin resistance indices, lower inflammation, better clearance of medium-sized molecules such as β2-microglobulin, improved nutrition, and better quality of life are other favorable outcomes associated with the presence of RKF [6-10].

Kalantar-Zadeh et al. [11] have proposed criteria for selecting appropriate iHD candidates, including Kru >3 ml/min/1.73m^2^ and urine output >500 ml/day, along with additional clinical parameters. However, there is a lack of standardized, well-developed protocols for implementing iHD in routine clinical practice. Safe implementation of iHD requires frequent RKF measurement and dialysis prescription adjustment.

The KDOQI Clinical Practice Guideline for Hemodialysis Adequacy [12,13] recommends measuring native kidney function as Kru and combining with the dialyzer clearance to determine the total effective small-solute clearance. The recommendation for the use of Kru to measure RKF is founded on the intrinsic underestimation of Kru and inherent protection provided to the patient when dosing dialysis. These guidelines allow for reduced dialysis dosing in patients with significant RKF (Kru ≥ 2 ml/min/1.73 m^2^).

While factors affecting kidney function in chronic kidney disease are well established, less is known about the impact of these factors on RKF decline after transitioning to hemodialysis. Patient demographics, comorbidities, medications, and dialysis prescription characteristics may all influence the rate of RKF loss [14].

Given the importance of maintaining RKF in hemodialysis patients, our study aims to analyze predictors of accelerated RKF loss in patients undergoing iHD. This research will contribute to the development of evidence-based strategies for optimizing iHD protocols and preserving RKF in ESRD patients.

## Materials and methods

Study design and participants

We conducted a retrospective cohort study analyzing 37 incident ESRD patients who initiated twice-weekly iHD in the outpatient setting from March 2017 to May 2022. Inclusion criteria for this incremental regimen were age ≥ 18 years, Kru ≥3 ml/min/1.73 m^2^, urinary output (UO) ≥ 500 ml/day, and ability to comply with monthly interdialytic urine collection. We excluded patients with severe to moderate left ventricular systolic dysfunction or active cancer.

Hemodialysis program

Patients were treated on a twice-weekly schedule, with a duration between 3 and 4 hours. All patients started on a low-flux hemodialysis and then switched to high-flux hemodialysis or hemodiafiltration according to the RKF loss. We used biocompatible polysulfone membranes, ultrapure water, and bicarbonate-buffered dialysis fluid in all treatments. Adequacy targets were standard Kt/V (Std Kt/V) of 2.3 volumes per week with a minimum delivered dose of 2.1 volumes, using a method of calculation that includes the contributions of ultrafiltration and residual kidney function, as suggested by the KDOQI Clinical Practice Guideline for Hemodialysis Adequacy 2015 [13]. Because of that, we used Std Kt/V, based on the normalizing generation rate of urea nitrogen to average weekly pre-dialysis urea, proposed by Gotch [15] and refined by Daugirdas et al. [16]. The assessment of the key urea kinetic parameters was done by using SPEEDY - Spreadsheet for the Prescription of incrEmental haEmoDalYsis [17]. To estimate Kru, we used a 24-hour urine sample collected during the day before the first hemodialysis treatment session of the month, after the biggest interdialytic period. Since we only collect serum urea pre- and post-dialysis from the session immediately following the collection period, we used the prediction equation developed by Daugirdas [18] to estimate the time-averaged concentration of serum urea nitrogen during those predialysis urine collection periods. Adequacy targets were evaluated every month. If necessary, to meet minimum targets or to prevent/treat hyperkalemia, hyperphosphatemia or fluid overload, the dialysis dose was adjusted including adjustment of membrane surface area, blood flow, bath flow, and dialysis duration.

Patients in iHD were switched to conventional HD (cHD) when their Kru falls below 2 ml/min/1.73 m^2 ^in two determinations and/or they develop uncontrolled hypertension, unmanageable fluid overload, adequacy problems (acidosis, hyperkalemia, hyperphosphatemia, and/or low clearance), and other clinical events that indicate that the number of hemodialysis sessions should be increased. Volume control was managed with additional support by multifrequency electrical bioimpedance (BCM®, Fresenius Medical Care, Bad Homburg vor der Höhe, Germany) monthly and on-demand evaluations. A maximum ultrafiltration rate of 10 ml/kg/h was determined.

Data collection

We extracted data from electronic medical records and paper charts. Baseline characteristics included socio-demographic data (age, sex, ethnicity, and body mass index), comorbidities (arterial hypertension, diabetes, left ventricular hypertrophy, coronary artery disease and peripheral vascular disease), Charlson Comorbidity Index, and etiology of renal disease (assumed based on clinical data, including histological renal results, when kidney biopsy was realized). We collected laboratory parameters and dialysis-related data at baseline and monthly thereafter. Intradialytic complications were also recorded.

Outcome definition

To evaluate predictors of accelerated loss of RKF, we defined accelerated loss of RKF as a decrease in Kru of at least 25% from baseline in the first three months. Patients were categorized into two groups: those with and without accelerated loss of RKF.

Statistical analysis

Baseline characteristics were summarized using descriptive statistics. Continuous variables were reported as mean ± standard deviation (SD) or median (interquartile range) depending on their distribution, as assessed by the Shapiro-Wilk test. Categorical variables were presented as frequencies and percentages. To compare patients with and without accelerated loss of RKF, we used independent samples t-tests or Mann-Whitney U tests for continuous variables, and chi-square tests or Fisher's exact tests for categorical variables, as appropriate. We identified potential predictors of accelerated loss of RKF based on previous studies. Variables with p-values ≤ 0.05 in univariate analysis were included in the multivariate logistic regression model. To avoid overfitting, we limited the number of variables according to the rule of at least 10 events per predictor variable. We used a stepwise selection method to determine the final model, considering both statistical significance and clinical relevance. For each predictor in the final model, we calculated odds ratios (OR) with 95% confidence intervals (CI). All statistical analyses were performed using SPSS version 29.0 (IBM Corp., Armonk, NY, USA), with a two-tailed p-value < 0.05 considered statistically significant. The follow-up period for each patient extended from iHD initiation until the transition to cHD, loss to follow-up, or the end of the study period (May 2022), whichever came first.

## Results

The baseline characteristics of the 37 patients enrolled are presented in Table 1.

**Table 1:** Baseline characteristics of iHD patients. ADPKD: Autosomal Dominant Polycystic Kidney Disease; ESRD: End-stage renal disease; GFR: Glomerular filtration rate; SD: Standard deviation; iHD: Incremental hemodialysis. GFR was estimated based on the average of the urea and creatinine clearance and corrected to 1.73 m^2^ body surface area.

Demographics	
Age, years (mean ± SD)	58 ± 13
Male gender (%)	54.1
Caucasian ethnicity (%)	97
Body mass index, kg/m^2^ (mean ± SD)	25 ± 4
Primary cause of ESRD (%)	
Diabetic nephropathy	32.4
ADPKD	16.2
Chronic glomerulonephritis	16.2
Hypertensive nephrosclerosis	13.5
Comorbid conditions (%)	
Arterial hypertension	89.2
Left ventricular hypertrophy	56.8
Diabetes	40.5
Coronary artery disease	10.8
Peripheral vascular disease	8.1
Charlson comorbidity index (mean ± SD)	4.7 ± 2.2
Hemodialysis vascular access (%)	
Arteriovenous fistula	54.1
Central venous catheter	45.9
GFR, mL/min/1.73 m^2^ (mean ± SD) [minimum; maximum]	7.5 ± 2.3 [3.5; 12.7]
Urine volume > 1000 ml/24 h (%)	93.3

Their mean age (± SD) was 58 ± 13 years; 54.1% (n=20) were male, and 97.0% (n=37) were Caucasian. The most common causes of ESRD were diabetic nephropathy (32.4%, n=12), followed by autosomal dominant polycystic kidney disease (16.2%, n=6), and chronic glomerulonephritis (16.2%, n=6). A large proportion of patients were diagnosed with arterial hypertension (89.2%, n=33), diabetes (40.5%, n=15), left ventricular hypertrophy (56.8%, n=21) and coronary artery disease (10.8%, n=4). The mean (± SD) Charlson Comorbidity Index was 4.7 ± 2.2. At the time of dialysis initiation, 54.1% (n=20) of patients had an arteriovenous fistula, and 45.9% (n=17) had a tunneled dialysis catheter as their primary vascular access. The initial glomerular filtration rate (GFR), calculated as the mean of urea and creatinine clearance and corrected for body surface area, was 7.5 ± 2.3 ml/min/1.73m^2^. Daily urinary output exceeding 1000 ml was observed in 93.3% of patients (28 out of 30 patients with available data).

From the initial cohort of 37 patients, complete data for evaluating accelerated loss of RKF was available for 30 patients (81.1%). Among these patients, nine (30%) experienced accelerated loss of RKF. The remaining 21 patients (70%) did not exhibit accelerated RKF loss during the first three months. Table 2 presents the comparison of baseline characteristics and clinical parameters between these groups.

**Table 2:** Characteristics among patients with and without accelerated RKF loss at three months. ADPKD: Autosomal Dominant Polycystic Kidney Disease; BMI: Body mass index; GFR: Glomerular filtration rate; IWG: Interdialytic weight gain; NHW: Normohydrated weight; SD: Standard deviation; UF: Ultrafiltration; RKF: Residual kidney function ^a^Arteriovenous fistula as initial vascular access to hemodialysis.^ b^GFR was estimated based on the average of the urea and creatinine clearance and corrected to 1.73 m^2^ body surface area. ^c^Intradialytic complications at the first month include symptomatic hypovolemia (cramping) and/or hypotension events (defined as a drop of systolic blood pressure greater than 25% of initial value or systolic blood pressure lower than 90 mmHg that required rescue fluid supplementation). Normohydrated weight was based on the results of the BCM measurements.

	Accelerated RKF loss (n=9)	No accelerated RKF loss (n=21)	p-value
Baseline characteristics			
Age, years (mean ± SD)	68.5 ± 2.2	55.6 ± 2.9	0.01
Female gender (%)	55.6	52.4	0.87
BMI > 25 kg/m^2^ (%)	44.4	42.9	0.90
History of diabetes or diabetic nephropathy (%)	77.8	33.8	0.04
ADPKD (%)	22.2	19	0.80
Chronic glomerulonephritis (%)	0	23.8	0.10
Arterial hypertension (%)	78	90	0.30
Left ventricular hypertrophy (%)	66.7	52.4	0.47
Coronary artery disease (%)	11.1	9.5	0.89
Peripheral vascular disease (%)	0	9.5	0.33
Charlson Comorbidity Index > 6 (%)	66.7	23.8	0.04
Arteriovenous fistula^a^ (%)	55.6	57.1	0.90
Urine volume, ml/24h (mean ± SD)	1701 ± 95	1868 ± 192	0.42
GFR^b^, ml/min/1.73m^2 ^(mean ± SD)	6.3 ± 0.8	8.6 ± 0.6	0.02
Proteinuria > 3.5 g/24h (%)	50	43.8	0.70
Serum phosphorus > 5.5 mg/dl (%)	44	55	0.59
Serum potassium > 5.5 mmol/l (%)	22.5	9.5	0.34
Epoetin alfa usage (%)	66.7	55	0.55
C-reactive protein > 5 mg/l (%)	50	50	1.00
1 month of iHD			
Intradialytic complications^c^ (%)	66.7	28.6	0.05
Dry-weight exceeding NHW > 1 kg (%)	57.1	12.5	0.03
3 months of iHD			
IWG > 3% of dry-weight (%)	42.8	55.6	0.50
UF rate within the first 3 months > 8 mL/kg/h (%)	66.7	47.6	0.30
Mean UF rate (± standard deviation) in the first 3 months, mL/kg/h	8.1 ± 0.7	7.8 ± 0.7	0.41

Patients with accelerated RKF loss were significantly older (mean age 68.5 ± 2.2 vs 55.6 ± 2.9 years, p=0.01) and had a higher prevalence of diabetes (77.8% vs 33.8%, p=0.04) compared to those without accelerated loss. A significantly higher proportion of patients with accelerated loss of RKF had a Charlson Comorbidity Index > 6 (66.7% vs 23.8%, p=0.04). They also exhibited a lower baseline GFR (6.3 ± 0.8 vs 8.6 ± 0.6 ml/min/1.73 m^2^, p=0.02).

During the first month of hemodialysis, patients with accelerated RKF loss experienced more intradialytic complications, including symptomatic hypovolemia and/or hypotension events (66.7% vs 38.6%, p=0.05) compared to those without accelerated loss. Hypotension events were defined as a drop of systolic blood pressure greater than 25% of the initial value or systolic blood pressure lower than 90 mmHg that required rescue fluid supplementation. In a significantly higher proportion of patients with accelerated loss of RKF, the dry weight prescribed was exceeding normohydrated weight (NHW) on BCM in more than 1 kg (57.1% vs 12.5%, p=0.03). No significant differences were observed between the groups in terms of gender distribution, body mass index, other comorbidities, type of vascular access or daily UO. Laboratory parameters (proteinuria > 3.5 g/24h, serum phosphorus > 5.5 mg/dl, serum potassium > 5.5 mmol/l, C-reactive protein > 5 mg/l) and epoetin alfa use at baseline were also not significantly different. Interdialytic weight gain and ultrafiltration rate within the first three months did not significantly differ between patients with and without accelerated RKF loss.

In the multivariate logistic regression analysis (Table 3), we examined five potential predictors of accelerated RKF loss: age > 70 years, history of diabetes or diabetic nephropathy, Charlson Comorbidity Index > 6, baseline GFR below 7 ml/min/1.73 m^2^, and intradialytic complications within the first month. Our analysis revealed that only baseline GFR below 7 ml/min/1.73 m^2 ^was an independent predictor of accelerated RKF loss (OR 25.41, 95% CI 1.22 - 530.27, p=0.04). The other variables did not show statistically significant associations with accelerated RKF loss in this model.

**Table 3:** Multivariate logistic regression analysis of predictors for accelerated RKF loss. CI: Confidence intervals; GFR: Glomerular filtration rate. GFR was estimated based on the average of the urea and creatinine clearance and corrected to 1.73 m^2^ body surface area.

Variable	Odds Ratio (Exp(B))	95% CI	p-value
Age > 70 years	2.23	0.16 – 30.1	0.55
History of diabetes or diabetic nephropathy	19.13	0.45 – 807.36	0.12
Charlson Comorbidity Index > 6	0.41	0.02 – 10.22	0.59
GFR < 7 mL/min per 1.73m^2^	25.41	1.22 – 530.27	0.04
Intradialytic complications within first month	0.60	0.03 – 12.64	0.74

At the end of follow-up, 17 patients (45.9%) transitioned to cHD, with 43% (n=16) making this transition within the first six months. Factors associated with the early transition to cHD (within the first six months) included higher ultrafiltration rates during the first month (7.1 ± 3.4 vs 5.0 ± 2.0 ml/kg/h; p=0.03) and more frequent intradialytic complications in the first trimester, including symptomatic hypovolemia and/or hypotension (58% vs 18%; p=0.026). The median time on iHD was six months (IQR 1-34 months). Additionally, two patients switched to peritoneal dialysis (PD), and two patients received a kidney transplant. One patient died during a surgical procedure.

## Discussion

Our study on 37 incremental hemodialysis patients revealed that 30% (n=9) experienced accelerated loss of RKF within the first three months of treatment. There is no standard definition of a rapid decline in RKF. We therefore defined accelerated loss of RKF as a decrease in Kru at three months above 25% from the baseline. Previous studies reported decline rates ranging from 5.8% to 7% per month in HD patients [19,20]. Assuming the upper limit of 7% decline per month, this would result in a 21% decline over three months. Consequently, we selected a 25% decline as indicative of an accelerated decline. We chose the three-month cut-off period since previous studies showed that the decline in RKF was most pronounced during the first three months after the start of treatment [21,22]. Although creatinine-based equations such as the Chronic Kidney Disease Epidemiology Collaboration (CKD-EPI) are recommended for estimating GFR in clinical practice, they have reduced performance in advanced stages of kidney disease [23]. We calculated GFR using the average of creatinine and urea clearances, corrected to 1.73 m^2^ body surface area, due to its superior performance at lower GFR levels, as suggested by the 2005 European Best Practices Guidelines [24]. This superiority compared to equations that only use serum creatinine can be explained by the relative preservation of tubular functions (including tubular secretion of creatinine and reabsorption of urea) at reduced levels of GFR [25]. The main challenge could be the 24-hour urine collection. However, since our patients will need to collect monthly 24-hour urine samples during enrollment in the iHD program, we believed that good compliance with this baseline collection for GFR calculation would help us determine if the patient was a suitable candidate for this hemodialysis schedule.

Patients with accelerated RKF loss were generally older, had a higher comorbidity burden, a higher prevalence of diabetes, and experienced more intradialytic complications.

While not statistically significant in the multivariate analysis, the older age and increased comorbidities in the accelerated RKF loss group warrant attention. Regarding age, there are conflicting results. Obi et al. [26] and He et al. [27] showed that older age at baseline was associated with a faster decline in Kru. However, Moist et al. [28] did not find an association between increasing age and RKF loss. Aging is associated with alterations in renal vasculature, including arterial stiffening and reduced renal blood flow. These changes could make older people more vulnerable to the rapid fluid and solute shifts during hemodialysis, which can cause hemodynamic stress, potentially accelerating the loss of remaining kidney function. Additionally, the hyperfunctioning of remaining nephrons could have a reduced effect on residual renal clearance due to the decreased nephron mass associated with age. Our finding of an association between a decrease in RKF and the Charlson Comorbidity Index has also been reported in the literature [29]. Like aging, comorbidities could contribute to the overall frailty and susceptibility of patients to rapid RKF decline.

The higher prevalence of history of diabetes or diabetic nephropathy was also associated with accelerated RKF loss. This association has been previously reported in the literature [26], with some studies showing a predictive value for RKF loss (particularly in PD patients) [28]. This faster rate of decline has been observed to begin during pre-dialysis CKD and continue into the early stages of hemodialysis treatment in incident patients [30].

The higher incidence of intradialytic complications, particularly symptomatic hypovolemia and hypotension, in patients with accelerated RKF loss highlights the importance of careful fluid management in preserving residual function. Our observation is consistent with other studies [15,30] which also found that intradialytic hypotension contributes to the decline of RKF. Hypotension episodes could reduce blood perfusion in remnant nephrons, leading to ischemic kidney damage and accelerating the loss of RKF. The significantly higher proportion of patients with accelerated RKF loss who had a dry weight prescribed exceeding NHW by more than 1 kg may be due to their diminishing capacity to adjust urine volume according to hydration status, ultimately leading to hypervolemia. Currently, there are opposing opinions regarding the best fluid management for patients requiring dialysis. While the contraction of extracellular volume accelerates the loss of RKF, expansion can also have undesirable effects on arterial hypertension and left ventricular hypertrophy. The objective would be to achieve an equilibrium of extracellular volume that would allow for preserving RKF but without provoking the negative effects of volume overload.

The finding that a baseline GFR below 7 ml/min/1.73 m^2^ is an independent predictor of accelerated RKF loss (OR 25.41, 95% CI 1.22-530.27, p=0.04) should be interpreted carefully. The cut-off value of GFR < 7 ml/min/1.73 m^2^ was determined based on our statistical analysis and clinical relevance. In our univariate analysis, we found a significant difference in baseline GFR between patients with and without accelerated loss of RKF (6.3 ± 0.8 vs 8.6 ± 0.6 ml/min/1.73 m^2^, p=0.02). We chose 7 ml/min/1.73 m^2^ as the cut-off point as it approximates the midpoint between these two values. Limited data exists analysing the association between baseline GFR and the rate of RKF loss, particularly in hemodialysis patients. Moist et al. observed that incident peritoneal dialysis (PD) patients with higher estimated GFR at ESRD initiation had a lower risk of RKF loss at one-year follow-up; however, they did not find the same association among hemodialysis-treated patients [28]. If a lower baseline GFR results from an accelerated decline in the pre-dialysis phase, this trajectory may continue after dialysis initiation, leading to more rapid RKF loss. In incident incremental hemodialysis patients, since dialysis prescription depends on RKF, those with an accelerated trend in GFR loss in the pre-dialysis setting may be at risk for underdialysis. Consequently, patients with a baseline GFR below 7 ml/min/1.73 m^2^ may benefit from more intensive monitoring and potentially earlier interventions to preserve RKF. Our findings should not encourage the early initiation of hemodialysis but rather help identify suitable candidates for iHD programs.

We acknowledge several limitations in this study. First, the small sample size and single-center nature of the study limit the generalizability of our findings. However, our iHD program had restricted criteria for patient selection, which could compromise the applicability of our results to other iHD regimens. Second, the study was retrospective and observational, and it is possible that the trajectory of RKF decline was already present in the pre-dialysis setting. Third, the wide confidence interval in the multivariate analysis for baseline GFR as a predictor suggests the need for larger studies to confirm this association. Nevertheless, our study focused specifically on iHD patients, providing valuable insights into this growing population. Larger, multi-center studies are needed to validate these findings and potentially identify additional predictors of accelerated RKF loss in iHD patients. Additionally, prospective studies will be crucial to examine the impact of interventions targeting modifiable risk factors, such as fluid management strategies, on RKF preservation.

## Conclusions

In conclusion, our study identifies baseline GFR as a key predictor of accelerated RKF loss in iHD patients. The findings highlight the importance of careful patient selection and management in iHD programs. By identifying patients at higher risk of rapid RKF decline, clinicians can implement targeted strategies to preserve RKF and potentially improve long-term outcomes.
